# Optical Excitation of Converging Surface Acoustic Waves in the Gigahertz Range on Silicon

**DOI:** 10.3390/s22030870

**Published:** 2022-01-24

**Authors:** Andrey Y. Klokov, Vladimir S. Krivobok, Andrey I. Sharkov, Nikolay Y. Frolov

**Affiliations:** 1 P.N. Lebedev Physical Institute of the Russian Academy of Sciences, 53, Leninskiy Prospekt, 119991 Moscow, Russia; kolob7040@gmail.com (V.S.K.); ashrk@yandex.ru (A.I.S.); 2 Institute for Laser and Plasma Technologies, Moscow Engineering Physics Institute, National Research Nuclear University MEPhI, 31 Kashirskoe Shosse, 115409 Moscow, Russia; frolil199999@gmail.com

**Keywords:** surface acoustic waves, converging wave, dynamic deformation, pump–probe technique

## Abstract

The optical excitation and propagation of converging surface acoustic waves on silicon with orientations (001) and (111) have been experimentally studied. An axicon-assisted formation of an annular irradiated region on the sample surface served as a source for converging surface waves. Surface wave patterns at different times were recorded using a Sagnac interferometer with spatial resolution. A study of the field distribution at the focus showed that, in spite of elastic anisotropy, which generally leads to aberrations, the acoustic energy can be concentrated into a spot with dimensions close to the diffraction limit. An asymmetric excitation distribution makes it possible to control the structure of the converged wave field at the focus, providing an effective tool for all-optical diagnostics of the local crystal structure as well as electronic properties of quantum objects embedded in the solid-state matrix.

## 1. Introduction

Recently, the property management of quantum systems by means of coherent phonons—deformation pulses—has been actively studied. For example, a number of studies have demonstrated the possibility of managing the luminescence properties and the spin subsystem of color centers in diamond, as well as the microcavity radiation [1,2,3,4].

In most of these experiments, the dynamic deformation value is at the level of 10^−4^–10^−5^. In terms of a crystal lattice or a bonds configuration near a lattice defect, such a value of dynamic deformation is small and its effect on the electron system can be considered within the framework of the standard perturbation theory. It is of interest to study the electron–phonon interaction within the limit of large local deformations, when perturbation theory is inapplicable. Moreover, it is very important to research irreversible structural changes caused by an intense elastic wave, such as the generation of local defects [5] and rearrangement/recharge of already existing structural defects [6]. To implement controlled dynamic deformations, both bulk and surface elastic waves can be used. Surface acoustic waves (SAW) that penetrate to a depth of the order of the wavelength allow to study the interaction with quantum objects located on the sample surface or within close proximity to it.

There are two commonly used methods of SAW excitation, the first is based on the piezoelectric effect, and the second is optical, based on elastic stresses excitation upon laser radiation absorption. Generating pulses of large deformation by piezoelectric methods requires the creation of complex shape interdigital transducers (IDT), which provide SAW focusing [7,8,9]. An obvious disadvantage of such methods is the complexity of matching the region of large SAW amplitude with the investigated area of the quantum object. Moreover, IDTs do not allow the broadband SAWs formation. In addition, the main requirement for the materials is to have piezoelectric properties which, in turn, creates an obvious restriction for the sample choice. As the sample is non-piezoelectric, it needs additional piezoelectric film deposition on a non-piezoelectric substrate.

Optical laser methods of SAW excitation are undoubtedly much more convenient, as they enable to obtain SAWs with a spectral width limited by laser beam focusing, reaching several GHz [10]. The SAW pulses amplitude upon optical excitation is limited by the sample damage threshold. However, recently, due to the use of an annular rather than point or linear sample irradiation, it has become possible to significantly weaken the limitations associated with the damage threshold, and to obtain converging SAWs. In particular, optical excitation of converging SAW on glass allowed to achieve an acoustic breakdown in the center of the ring [11,12]. In this case, the generation and propagation of the SAW occurred in a mode close to linear, with the exception of the region close to the focus.

In the experiments, the formation of an annular intensity distribution on the sample surface is easily achieved using a conical lens—an axicon [13]. For elastically anisotropic materials, such excitation is not optimal and can lead to aberrations in the vicinity of the focus. For this reason, the success of using ring excitation on anisotropic materials to achieve large dynamic deformations is not obvious. Nevertheless, we assumed that if the distance from the excitation region to the focus is not too large, the dephasing of elementary waves caused by the angular dependence of the group velocity will be insignificant and it will be possible to focus the acoustic energy into a small spot. By modifying the ring excitation (for example, by covering part of the ring with a small screen), it is possible to change the structure of the field at the focus. Regarding amorphous glasses, such rearrangement was demonstrated in [14].

In the case of a crystal, due to elastic anisotropy, a new possibility for controlling the field structure at the focus can arise. Choosing the screen position orientation with respect to the crystallographic axes of the sample SAW polarization can be changed in the focus area. Despite the existence of relevant works on amorphous materials [11,12,13,14], the possibility of achieving the sharp focusing of SAW for anisotropic materials, as well as the possibility of controlling the field polarization at the focusing point, have not been discussed in the literature.

The aim of this work was to realize converged optically-excited SAWs on the surfaces of crystalline materials, the elastic properties of which, in contrast to amorphous glasses, reveals some extent of anisotropy. Silicon monocrystal wafers with surface orientations (001) and (111) were chosen as model materials.

A study of the field distribution at the center of converged waves showed that in each case the acoustic energy can be concentrated into a spot with dimensions close to the diffraction limit. At the same time, the implementation of asymmetric excitation enables to control the structure of the field at the focus, providing an effective tool for all-optical diagnostics of quantum objects embedded in crystalline materials.

## 2. Materials and Methods

**Figure 1:**
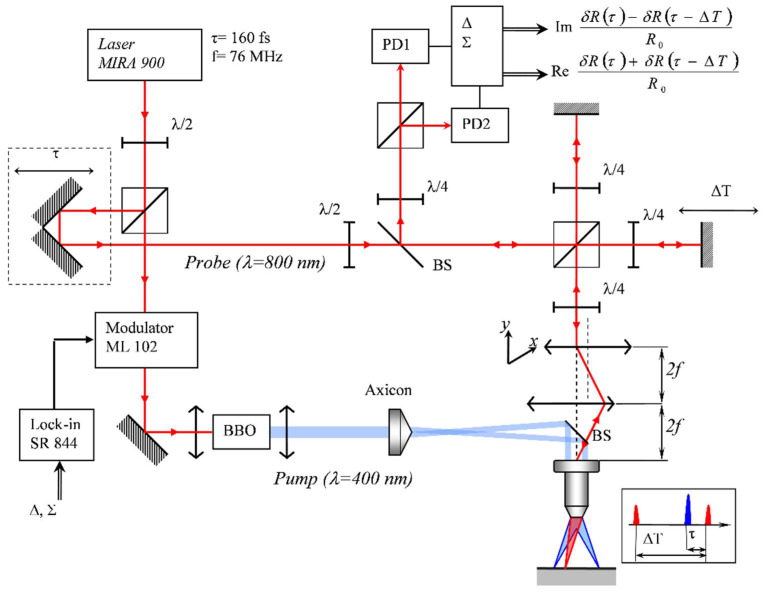
Experimental setup.

**Figure 2:**
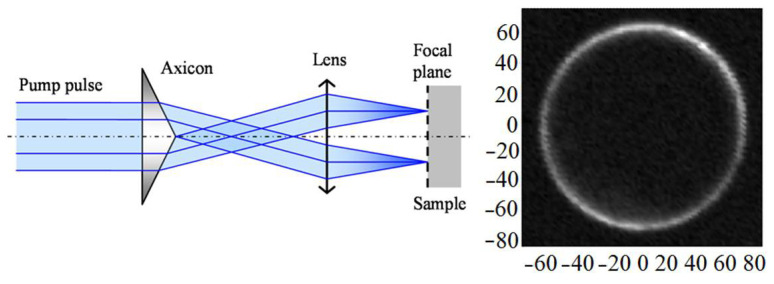
Left: scheme of an axicon application to form a ring excitation. Right: intensity distribution measured on the sample surface.

The samples were 0.8 mm thick silicon wafers oriented (001) or (111) covered by thermally deposited ~400 nm thick aluminium film. The experimental setup was based on the well-known pump–probe technique with a Mira-900 femtosecond laser (160 fs pulse width, 76 MHz repetition rate) as a source (Figure 1). The laser radiation was split into two beams. The first beam excited the sample after frequency doubling (wavelength 400 nm, pulse energy <1 nJ). An electro-optical modulator was installed in the excitation channel to implement synchronous detection. The second beam (wavelength 800 nm, pulse energy 0.05 nJ) registered changes in the optical reflectance. Surface waves were generated as a result of the thermoelastic effect under the laser pulse absorption. Propagating elastic waves caused surface oscillation, leading to a change in the reflection coefficient phase of the probe beam (~10^−6^–10^−4^), which was detected using a modified Sagnac interferometer [15]. The probe beam in this interferometer was divided into two channels, the reference and the probe, and the pulse from the reference channel hit the sample earlier than from the probe one, but both pulses arrived at the detector simultaneously. As a result of the reference and probe pulses interference and after synchronous detection of the detector output two signals were produced: one was proportional to the phase change $u_{\Delta }\sim \delta \varphi \left(\tau \right)-\delta \varphi \left(\tau -\Delta T\right)$ at times *τ* and *τ*−Δ*T*; another was proportional to the sum of the relative changes in the reflection coefficient modulus at times *τ* and *τ* − Δ*T* $u_{\Sigma }\sim \left|\frac{dR\left(\tau \right)}{R_{0}}\right|+\left|\frac{dR\left(\tau -\Delta T\right)}{R_{0}}\right|$. To scan the surface and register elastic waves, surface and bulk, a 4F scanner was introduced into the scheme. The optical delay line enabled to observe the patterns of the elastic field distribution at different times relative to the excitation pulse. In addition, an axicon could be installed in the excitation channel for ring sample excitation. We used axicons with angles of 0.5° and 1.0° and microscope objectives with a numerical aperture from 0.40 to 0.95. This enabled to form an annular intensity distribution on the sample surface with a 50–240 µm diameter and a 2–4 µm width. Figure 2 shows the diagram of the ray path during the ring excitation formation, as well as the experimentally measured intensity distribution on the sample surface (67 µm in radius, 4 µm in width). The energy irradiance was ~70 µJ/cm^2^, which was >10^3^ times less than the radiation resistance threshold of our samples.

## 3. Results and Discussion

### 3.1. Diverging SAW

First, it was necessary to determine the structure and types of surface waves that converge to a point on crystal surfaces with a given orientation. For this purpose, the inverse problem was solved; we investigated surface waves propagating along a given surface under point excitation. In these experiments, the laser spot size was d~2 µm, and the spectrum width of generated SAWs was estimated from above as Δf = V_R_/d~2 GHz, where V_R_ is Rayleigh wave velocity [10].

**Figure 3:**
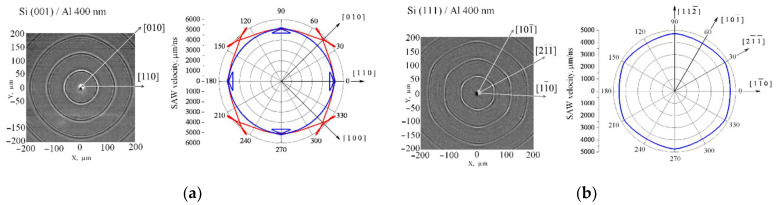
(**a**) Silicon sample with orientation (001); (**b**) Silicon sample with orientation (111). Experimentally registered diverging SAW on silicon surface covered by aluminium film (left). Calculated SAW group velocity (right).

Surface waves were detected under point excitation on silicon surfaces (001) and (111). In Figure 3a, the left picture shows the SAW pattern on the (001) surface where consecutive wavefronts correspond to repetitive laser pulses. The normal frequency dispersion associated with the presence of an aluminium film on the silicon surface begins to emerge in the form of contours following the main wavefront at >180 μm from the center. Despite the fact that fronts on Si (001) differ slightly from circles, they were formed by two modes. Calculations (Figure 3b, right picture) showed that one of the modes (blue curve) is Rayleigh in the [001] direction and equivalent ones and becomes slow shear in the [110] direction, while another one (red curve) is Rayleigh in the [110] direction and becomes a pseudosurface in the [010] direction. Similar measurements were carried out on silicon with the (111) orientation. The resulting patterns of SAW wave fronts are shown in Figure 3b, left picture. Calculations (Figure 3b, right picture) showed that these fronts correspond to Rayleigh waves. For both crystallographic orientations of silicon, the deviation of the group velocity from the isotropic case was less than 5%.

Thus, we found that a Rayleigh wave front close to the annular front is promising for achieving sharp focusing despite the anisotropy.

### 3.2. Ring Excitation

**Figure 4:**
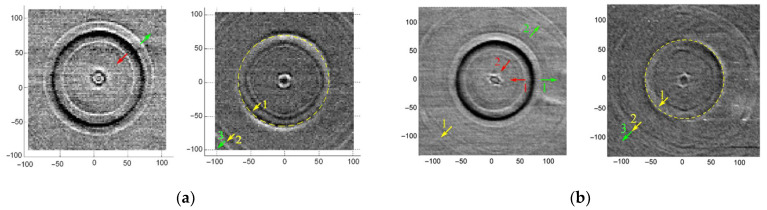
Converging SAW on silicon surface covered by aluminium film at different time delay. (**a**) Silicon sample with orientation (001); (**b**) Silicon sample with orientation (111).

Figure 4a shows the SAW patterns on silicon (001) with ring optical excitation. The excitation ring radius is 67 µm, which is slightly larger than the distance over which the SAW propagated during the laser pulse repetition period (~65 µm). The probe pulse was delayed relative to the exciting one by 3.46 ns. The dark ring in the left picture corresponds to the region heated by the (*n*th) laser pulse. Two annular fronts, marked by arrows, depart from this region: to the center of the excitation ring (marked by red arrow) and outwards (marked by green arrow). The fronts cover the distances (~17 μm) which correspond to a SAW velocity of ~4.9 μm/ns. In the ring center, there is the SAW front from the previous (*n*−1st) laser pulse, which has already passed the focal point and propagates from it.

It can be seen that, the wave front passing through the focus has a visible symmetry of the fourth-order, which corresponds to Si (001) symmetry. In contrast, the anisotropy of the converging wave has not yet become distinguishable. The diverging front from the *n*−2nd pulse is not visible since it falls into the excitation ring. In the corners there are traces from the SAW front excited by an *n*−3rd pulse.

The right picture in Figure 4a shows the SAW fronts before the arrival of the *n*th laser excitation. The dashed line indicates the position of the excitation ring. In the ring center, the SAW front from the previous (*n*−1st) laser pulse is visible; it is going to the focal point. Yellow arrow 1 marks the SAW front from *n*−2nd pulse, arrow 2—from *n*−3rd pulse. Green arrow 3 shows the SAW front from the *n*−1st pulse, which immediately propagates outward from the excitation region. This front has no noticeable anisotropy, which can be seen when scanning over a wider region, while all other fronts (1 and 2) have observable anisotropy corresponding to the fourth-order symmetry typical for Si (001).

A similar experiment on a silicon surface (111) is shown in Figure 4b. The left picture corresponds to the probe pulse 3.46 ns delay relative to excitation pulse. One can see a circular front, propagating towards the center from the excitation region (marked by red arrow 1), as well as a front leaving the excitation region (green arrow 1). The dark contours corresponds to the longitudinal leaky SAW (LLSAW), marked by red and green arrows 2, are faintly visible here. This front travels a distance of 28 µm in 3.46 ns, which corresponds to a velocity value of ~8.1 µm/ns. This velocity is close to that of a quasi-longitudinal wave, which, depending on the direction of propagation, is in the range from 8.30 to 9.35 µm/ns [16]. The near-field structure is visible in the center and corresponds to the leading edge of an (*n*−1st) pulse that has passed through the focal point. Its symmetry differs significantly from the crystal third-order symmetry and the SAW sixth-order symmetry natural for (111) silicon. The front from the (*n*−2nd) pulse is not visible since it is located under the excitation region, while the front from the (*n*−3rd) pulse (marked with yellow arrow 1) has an observable anisotropy similar to the fronts shown in Figure 3 when a SAW was excited at a point. The right picture of Figure 4b shows the converging SAW on silicon (111) when the delay between the exciting and probe pulses was approximately zero. Inside the excitation region, there is a contour in the form of a nut marked by a yellow arrow 1—this is the SAW front from (*n*−2nd) pulse. When departing from the excitation region, it formed a circle, like the front indicated by the red arrow 1 in the left picture, then it passed through the focal point, convert from converging to diverging, and on this wavefront the crystal symmetry has already manifested itself. At the initial moment, the crystal elastic anisotropy did not appear at the front of the surface wave, but after passing through the focal point, it clearly appeared.

### 3.3. Converging SAW

**Figure 5:**
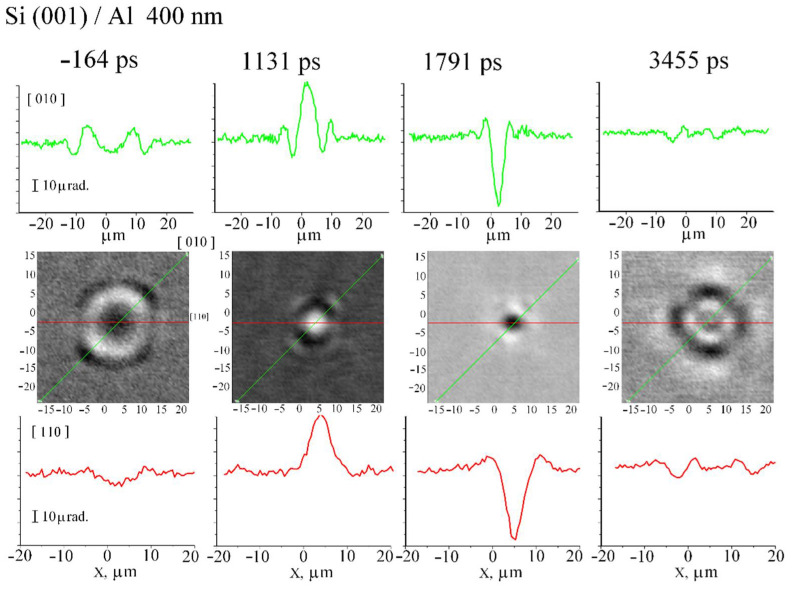
Center block of pictures: converging SAW field on silicon with orientation (001) covered by aluminium film at several time moments. Green lines indicate lateral direction [010], red lines indicate lateral direction [110]. Top block and bottom block of pictures show SAW profile along these lines, respectively.

**Figure 6:**
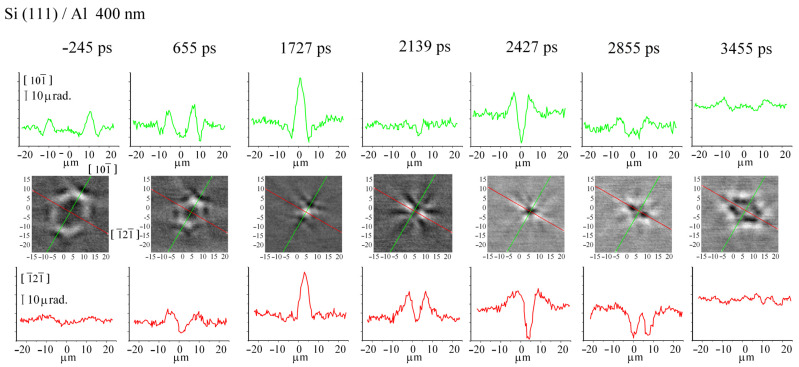
Center block of pictures: converging SAW field on silicon with orientation (111) covered by aluminium film at several time moments. Green lines indicate lateral direction $\left[10\bar{1}\right]$, red lines indicate lateral direction $\left[\bar{1}2\bar{1}\right]$. Top block and bottom block of pictures show SAW profile along these lines, respectively.

We investigated the surface wave distribution at the focus on both silicon (001) and silicon (111). Figure 5 shows SAW field distribution patterns in the vicinity of the focus on the silicon crystal (001). It can be seen that at all times the symmetry of the field distribution corresponds to the symmetry of the crystal. After focusing at 1.131 ns and 1.971 ns almost all of the acoustic energy is in the ~5–7 µm spot, close to the ~4–5 µm wavelength SAW, emitted by a ~4 µm ring. On the other hand, the asymmetric field pattern indicates strong aberrations on silicon with the (111) orientation (Figure 6). Nevertheless, SAW focusing into a 5–7 µm spot, also close to a 4–5 µm wavelength, is also observed at 1.727 ns and 2.427 ns. Estimates of the deformation in the focus give a value of ~10^−5^, while the radiation resistance limit is more than 3 orders of magnitude larger, which allows us to state that deformations of ~10^−2^ in the ~5 μm focal region are achievable. The pulse duration at the focus can be estimated from the time it takes for the elastic pulse to pass through the focal region, which is ~2.2 ns (see Figure 6).

### 3.4. Half-Ring Excitation

**Figure 7:**
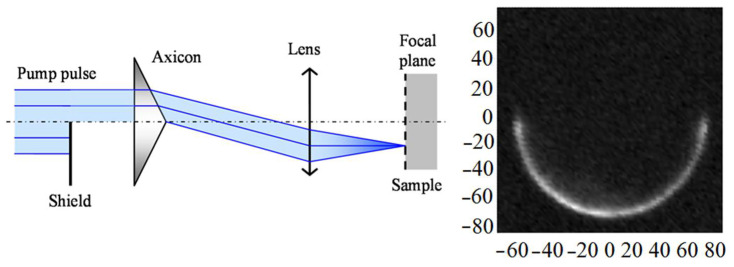
Left: scheme used to obtain a half-ring excitation. Right: intensity distribution measured on the sample surface.

We also measured the SAW field distribution in the vicinity of the focus on the (001) crystal under asymmetric excitation with a half ring. Figure 7 shows a diagram of such an excitation formation and the results of measuring the optical radiation intensity distribution on the sample surface.

**Figure 8:**
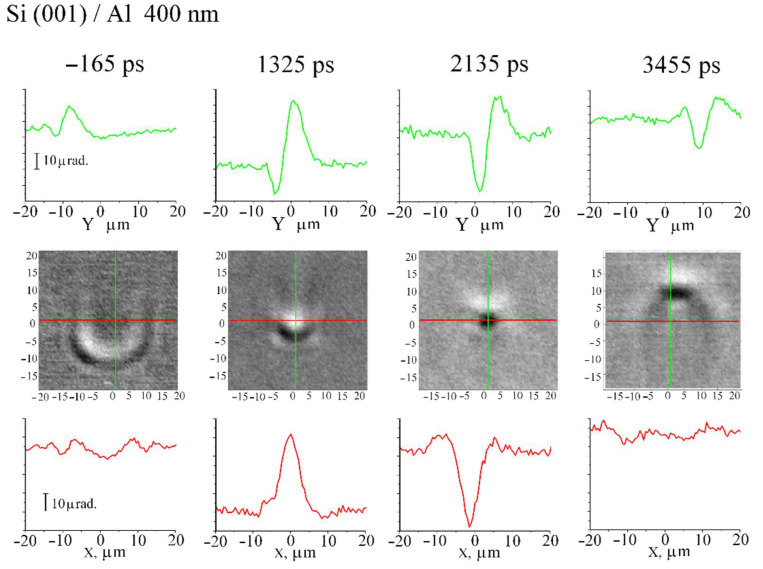
Center block of pictures: SAW field on silicon with orientation (001) covered by aluminium film at half-ring excitation at several time moments. Top block and bottom block of pictures shows SAW profile along green and red lines, respectively.

Pictures of the SAW field at different times are shown in Figure 8. Here, focusing into a ~7 μm spot is also observed; however, the field symmetry is even along the X-coordinate and odd along the Y-coordinate. Thus, changing the half-ring position relative to the sample crystallographic axes enables to control the structure of the field at the focus. Therefore, in spite of elastic anisotropy, surface waves are focused into a region (~5 μm) equal in order of magnitude to the diffraction limit.

So, from the given experimental results it follows that:

(1) A Rayleigh wave generated by circular optical excitation on the surface of crystalline materials can be focused into a spot close to the diffraction limit in size;

(2) The presence of SAW focusing enables to overcome the optical destruction limit of materials and to obtain >~10^−2^ deformation amplitudes by optical methods;

(3) The field structure of the surface wave allows the use of a flexible control mechanism based on the darkening of ring excitation fragments.

The listed above properties of converging surface waves make them a promising tool for a number of applications related both to the study of the structural properties of crystalline materials and to the implementation of a controlled effect on the electronic subsystem of quantum objects embedded in a crystalline matrix. In particular, the possibility of creating ~10^−2^ anisotropic deformation with a component along the converging wave plane, creates the prerequisites for a targeted action on dislocations and point defects. Thus, in optical experiments, it is possible to realize the controlled slip of dislocations and/or point defects diffusion. On the other hand, controlled anisotropic deformation should provide new methods for dynamical control of the properties of the luminescent centers located in crystal matrices, especially when taking into account the spin–orbit interaction. It should be noted that due to the possibility of obtaining high amplitudes, the potential scales of the electronic spectrum rearrangement are not achievable in conventional experiments with an external electric/magnetic field or static deformations.

## 4. Conclusions

We studied the excitation and propagation of converging SAWs on the silicon surface with the (001) and (111) orientations. On both surfaces, the SAW was found to be focused into a circle with dimensions ~5–7 μm on the order of the diffraction limit.

The use of circular optical excitation under experimental conditions enabled to minimize the requirements for sample radiation resistance by >1000 times and, in the future, achieve deformation in the focus ~10^−2^ without (optical) sample destruction.

Changing the excitation symmetry allows to control the SAW field distribution at the focus, which can be interesting when studying local crystal structure and/or the electronic properties of different systems.

The results obtained can be extended to other crystalline materials with elastic anisotropy similar (or less) to silicon. This is the case, for example, for diamond in which the anisotropy is less than that of silicon.

## Data Availability

Not applicable.
